# What is Threatening Forests in Protected Areas? A Global Assessment of Deforestation in Protected Areas, 2001–2018

**DOI:** 10.3390/f11050539

**Published:** 2020

**Authors:** Christopher M. Wade, Kemen G. Austin, James Cajka, Daniel Lapidus, Kibri H. Everett, Diana Galperin, Rachel Maynard, Aaron Sobel

**Affiliations:** 1 RTI International, 3040 E Cornwallis Rd, Durham, NC 27709, USA;; 2 RTI International, 3040 E Cornwallis Rd, Durham, NC 27709, USA;; 3 RTI International, 3040 E Cornwallis Rd, Durham, NC 27709, USA;; 4 RTI International, 3040 E Cornwallis Rd, Durham, NC 27709, USA;; 5 RTI International, 3040 E Cornwallis Rd, Durham, NC 27709, USA;; 6 US EPA, 1200 Pennsylvania Avenue, N.W., Washington, DC 20460, USA;; 7 Former ORISE Fellow for US EPA, 1200 Pennsylvania Avenue, N.W., Washington, DC 20460, USA;; 8 US EPA, 1200 Pennsylvania Avenue, N.W., Washington, DC 20460, USA;

**Keywords:** Protected Areas, deforestation, tree cover loss, global forest

## Abstract

The protection of forests is crucial to providing important ecosystem services, such as supplying clean air and water, safeguarding critical habitats for biodiversity, and reducing global greenhouse gas emissions. Despite this importance, global forest loss has steadily increased in recent decades. Protected Areas (PAs) currently account for almost 15% of Earth’s terrestrial surface and protect 5% of global tree cover and were developed as a principal approach to limit the impact of anthropogenic activities on natural, intact ecosystems and habitats. We assess global trends in forest loss inside and outside of PAs, and land cover following this forest loss, using a global map of tree cover loss and global maps of land cover. While forests in PAs experience loss at lower rates than non-protected forests, we find that the temporal trend of forest loss in PAs is markedly similar to that of all forest loss globally. We find that forest loss in PAs is most commonly—and increasingly—followed by shrubland, a broad category that could represent re-growing forest, agricultural fallows, or pasture lands in some regional contexts. Anthropogenic forest loss for agriculture is common in some regions, particularly in the global tropics, while wildfires, pests, and storm blowdown are a significant and consistent cause of forest loss in more northern latitudes, such as the United States, Canada, and Russia. Our study describes a process for screening tree cover loss and agriculture expansion taking place within PAs, and identification of priority targets for further site-specific assessments of threats to PAs. We illustrate an approach for more detailed assessment of forest loss in four case study PAs in Brazil, Indonesia, Democratic Republic of Congo, and the United States.

## 1. Introduction

**Figure 1.**
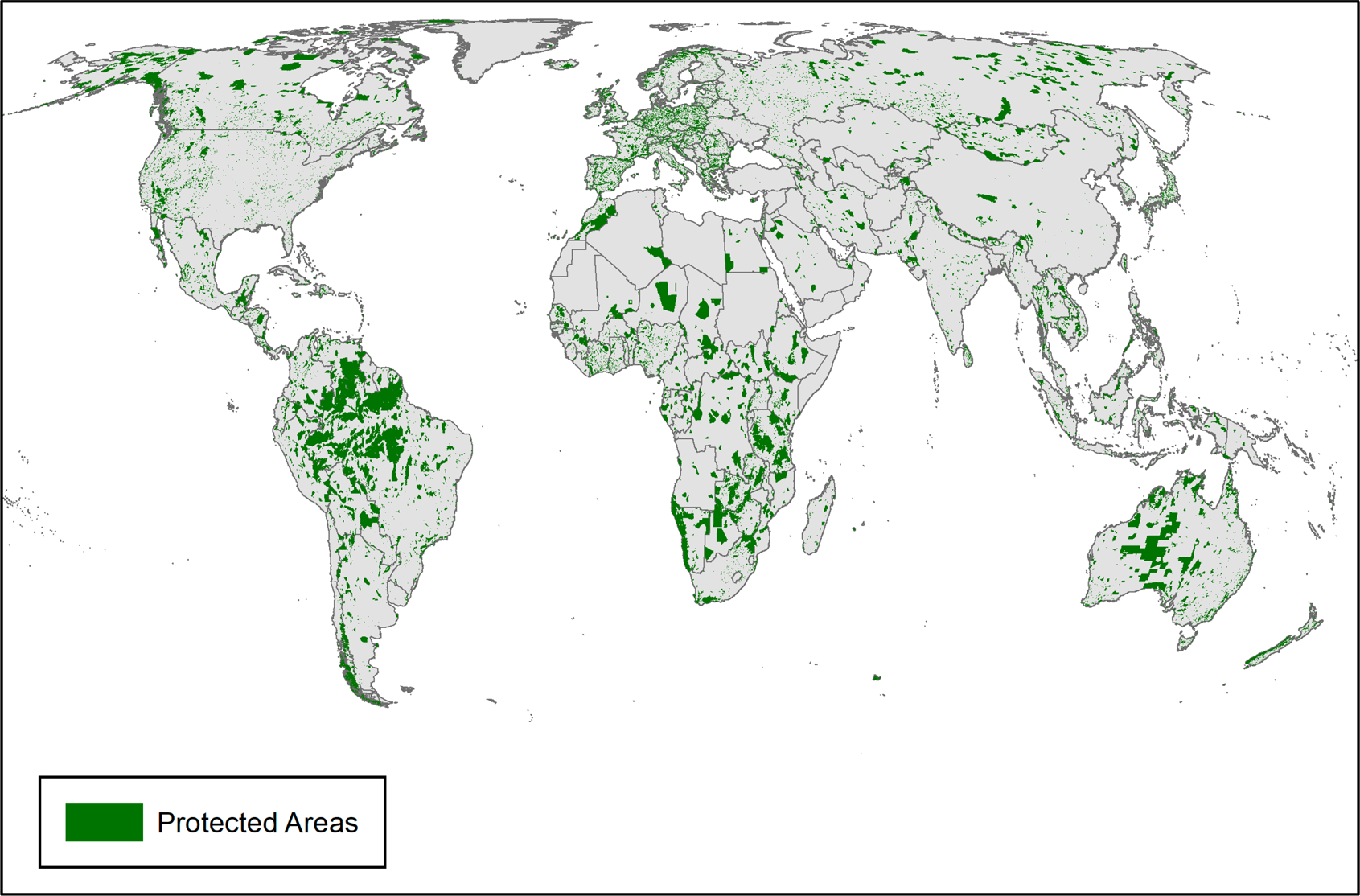
Location of Protected Areas (PAs) based on World database on Protected Areas [5].

Protected Areas (PAs) are a key strategy for safeguarding global biodiversity and ecosystem services. As of 2018, there were more than 230,000 terrestrial PAs worldwide, protecting 14.9% of the earth’s surface and inland waters outside of Antarctica [1], and 5.2% of global tree cover (Figure 1). The extent of PAs has increased substantially since the 1990s, and under the Convention on Biological Diversity, nations committed to further increasing the land area in PAs to 17% by 2020. Despite this, PAs are under increasing threat from anthropogenic activities, including encroachment for settlements, agriculture, mining, logging, and poaching and bushmeat hunting [2]. Worldwide almost one-third of PAs are under intense human pressure, determined as the combined influence of built environments, agriculture, human population, and transportation infrastructure [3]. Moreover, less than half of PAs are free of any human pressure, and this pressure has increased since the 1990s [4].

Globally, between 2001 and 2012, 3% of PA forests and 5% of all forests were converted to other land cover types [6]. While the lower rate of forest loss in PAs relative to the global average may suggest that PAs are effective in preventing some, if not all, forest loss, several studies have shown that PAs are preferentially located in areas that have a lower risk of deforestation [7–10]. The locations of PAs are biased towards areas with lower potential agricultural revenues and limited access, in order to minimize conflict with extractive industries and thus reduce the cost of acquisition and establishment [11]. Nevertheless, studies controlling for these confounding factors generally demonstrate that PAs do provide additional protection beyond what would have been expected in the absence of their designation [12,13].

Here, we examine global tree cover loss in PAs globally over 2001–2018, providing the most up-to-date report on forest conversion trends in PAs. Previous analyses of forest loss have been restricted to national or regional scales (e.g., References [8,14]), and/or have not been recently updated (e.g., Reference [7]). We highlight regions and countries where PAs are succeeding and failing to prevent forest loss, a proxy for their ability to safeguard intact habitats and protect other provisioning and regulating ecosystem services. Next, we examine the land cover following tree cover loss in PAs, and variations in follow-up land cover over space and time. In particular, we measured the magnitude (extent) of forest to agricultural land conversion in PAs, as agriculture has been shown to be a dominant driver of deforestation in the tropics [15,16] and globally [17]. Our analysis provides useful information about what may be causing forest loss in PAs and informs the development of PA management and enforcement strategies that are tailored to the agents of change on the ground. Our global assessment should be considered a screening tool to identify priority regions for further detailed investigation of threats to PAs.

## 2. Materials and Methods

To conduct our analysis, we took advantage of three recently published, or recently updated, spatially explicit datasets (Table 1), (1) protected areas from the World Database on Protected Areas (WDPA) [5], (2) 30 m resolution Global Forest Change (GFC) data representing tree cover loss annually, from 2001–2018 [18], and (3) 300 m resolution land cover maps for the years 2005, 2010, and 2015 from the European Space Agency–Climate Change Initiative (ESA-CCI) [19].

From the WDPA, we excluded marine PAs, PAs which have been proposed but not formally designated, and those without spatial information (e.g., only provided as point data). We examined forest loss trends in a given PA beginning the year after which it was formally designated, and in the ~10% of cases where the establishment year was not provided, we assumed that the PA had been established prior to 2001. We included all PA types in our analysis (Supplementary Table S1), including those designated in the International Union for Conservation of Nature (IUCN) categorization system as “Not Applicable”, “Not Assigned”, and “Not Reported”. These categories include some important types of protection, including lands managed by indigenous communities in Brazil and United Nations Educational, Scientific and Cultural Organization (UNESCO) Biosphere Reserves in Guatemala. However, these may be left uncategorized according to the IUCN typology due to reporting errors. We isolate trends in PAs which are more strictly protected according to the IUCN categorization system (strict nature reserves, wilderness areas, and national parks), in recognition of the fact that less stringent PA categories may support forest management and other types of sustainable land use change that would result in forest loss. Within IUCN category IV, it is recognized that active management, or modifications to the ecosystem (e.g., halting natural succession, providing supplementary food, or artificially creating habitats) will take place. Specifically, IUCN Category IV sites allow sustainable management of natural resources to maintain culturally defined ecosystems with unique biodiversity, but are not designed for industrial harvest levels [5], and Category VI areas allow the sustainable use of natural resources to promote ecosystem services. In the case where PA polygons overlapped, we assumed the most stringent level of protection. We converted the vector shapefile to a raster grid with spatial resolution of 30 m.

The GFC dataset [18] mapped tree cover loss, defined as the conversion from forest to non-forest, during the 2000–2018 period. We refer to this as forest loss, under the assumption that loss in PAs is predominantly natural forest loss as opposed to loss of planted trees or plantations. We restrict our analysis to areas with greater than or equal to 50% canopy cover in the year 2000 [18]. We tabulated forest loss through three time periods: 2001–2004, 2005–2009, and 2010–2014. We then categorized each forest loss pixel to a land cover class in the year immediately following each of these periods: 2005, 2010, and 2015, respectively. Previous research demonstrated that the land cover following forest loss does not change substantially in a 1–10-year period after the loss occurred. Our approach is based on a period of 1–4 years after forest loss [20]. For a given PA, we excluded any tree cover loss that occurred prior to the year of PA establishment. We additionally report loss in PAs from 2015 to 2018, though we cannot assign a follow-up land cover to this loss, as the most recent land cover map is from the year 2015.

We use the land cover type following forest loss to categorize the cause of deforestation. We acknowledge that subsequent land cover is only a proxy for the complex and dynamic causes of deforestation, but more detailed investigation of these underlying causes is not possible at the scale of our analysis. We reclassified the 22 land cover categories presented in the European Space Agency Climate Change Initiative (ESA-CCI) land cover dataset to seven categories (Supplementary Table S2) [21]. Our reclassification schema consolidated forest categories (e.g., broadleaf tree cover, needleleaf tree cover), shrubland categories (e.g., shrubland, mosaic herbaceous cover), grassland, and ‘other’ land cover types (e.g., urban, bare land, water bodies, snow cover). We retained three separate agriculture categories: cropland (including both rainfed and irrigated cultivated crops), mosaic cropland (>50% cropland mixed with trees, shrubs, and herbaceous cover), and mosaic vegetation (<50% cropland and >50% mixed trees, shrubs, and herbaceous cover). The mapped cropland land cover categories have reported accuracies of 73%–89%, except for the mosaic vegetation category. This land cover type has a reported accuracy of just 59%, largely due to commissions of the other cropland categories. Notably, ESA-CCIs agriculture category includes areas used for crop cultivation but does not include areas used for livestock grazing (pasture land or managed grasslands). ESA-CCI does include a grassland category, but does not differentiate natural grasslands from managed grasslands, as this is difficult at global scales using mid-resolution satellite imagery [22]. We resampled the landcover dataset to a 30 m pixel raster grid, matching the resolution of the forest loss map.

To gauge the robustness of our approach to classifying the land cover following forest loss using global-scale data, we compared our results to two previous studies which investigated drivers of deforestation using nationally and regionally specific datasets (Supplementary Table S3). We aggregated several land cover categories in order to facilitate comparison according to Supplementary Table S3. In Indonesia, the authors of Reference [15] found that about 67% of deforestation nationally was followed by agriculture and 40% of deforestation events in PAs were followed by agriculture. We found a similar proportion of forest loss to agriculture nationally (61%) and in PAs (38%). In South America, the authors of Reference [16] reported that 20% of deforestation was followed by agriculture and another 69% followed by pasture lands from 2001 to 2005. We estimated that 44% of forest loss was followed by agriculture, and another 44% was followed by grassland. The differences in Brazil may be partially explained by the challenge of differentiating natural grasslands from managed grassland or pastureland. The results of these robustness checks provide confidence that we can broadly track agriculture as a driver of forest loss using the global land cover dataset, though differentiating pastureland as a driver of forest loss remains difficult with currently available land cover maps (Supplementary Tables S4 and S5). We address the implications of this challenge in more detail in the Discussion Section.

We examined case studies of forest loss in PAs in four countries: Brazil, Indonesia, Democratic Republic of Congo, and the US (Supplementary Figure S1). We selected these countries because each has some of the highest forest loss globally and we wanted to include representation across continents and biomes (Supplementary Table S4). In each case, we investigated forest loss trends in a PA with one of the highest rates of forest loss nationally. We visualized forest cover and loss for each selected PA, examined the Landsat time series from 2000 to 2016 in Google Earth, and available high-resolution satellite imagery in the PA over the study period. We do not aim to provide a systematic validation of our approach to tracking forest loss and following land cover in PAs, but rather use these case studies to explore areas of concern in more detail with higher resolution satellite imagery.

## 3. Results

### 3.1. Ongoing Forest Loss in Protected Areas

**Figure 2.**
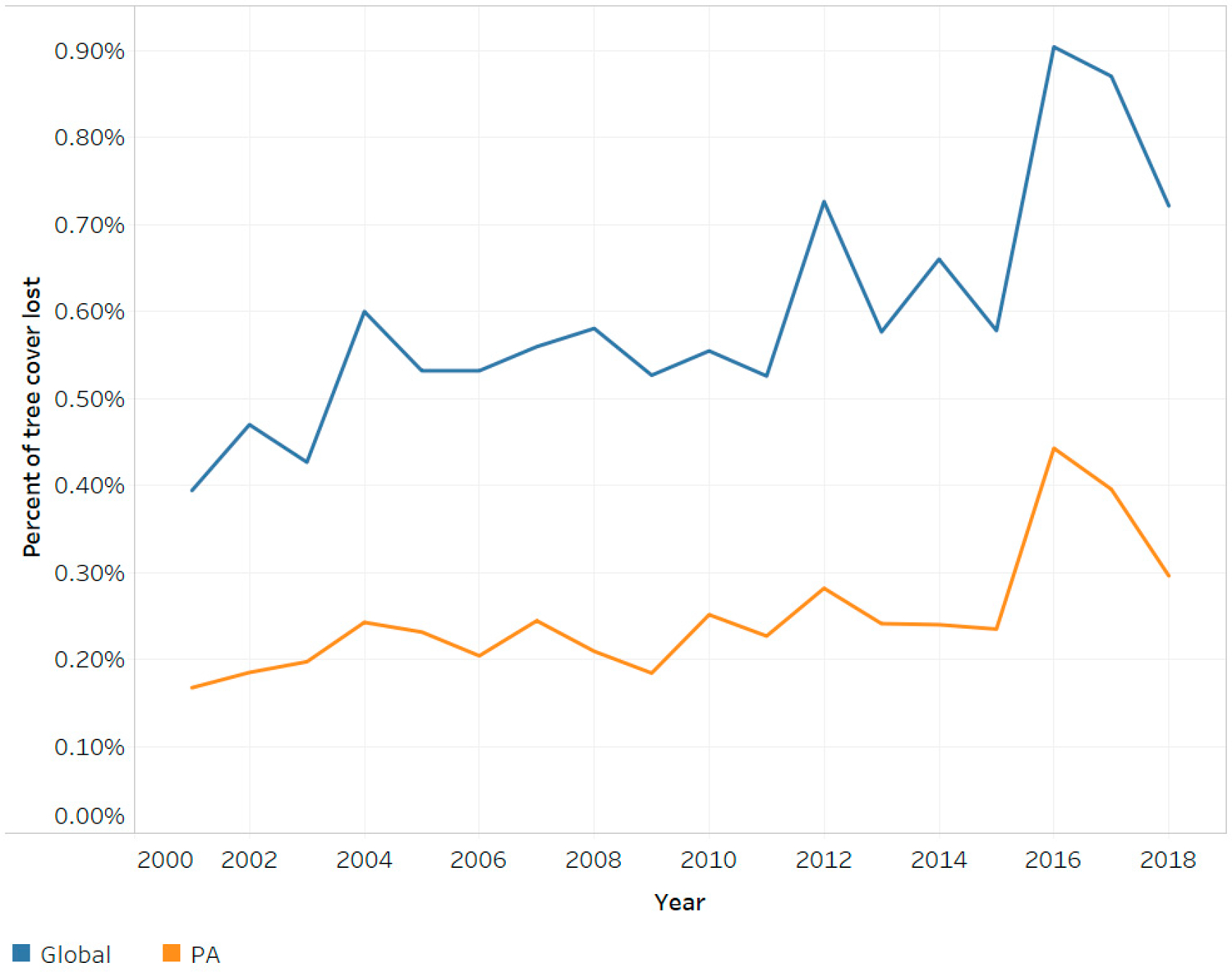
comparison of tree cover loss worldwide, and within PAs: 2001–2018 (% of tree cover lost). The distance between the global trend and PA trend is representative of (1) the effectiveness of PAs promoting natural resource conservation, and (2) the impact of location bias of PAs [9,11].

From 2001 to 2018, 12.2% of global forest area (401.3 million hectares (Mha) of 3289.4 Mha) and 4.1% of protected forest area (25.5 Mha of 628.1 Mha) experienced forest loss. Total forest loss generally increased continuously from 2001 to 2018, with a notable spike in 2016 likely due to a spike in forest fires [23]. Forest loss in PAs followed a strikingly similar trend, suggesting that PAs are not exempt from the underlying climatic and macroeconomic forces that drive forest loss globally (Figure 2).

**Figure 3.**
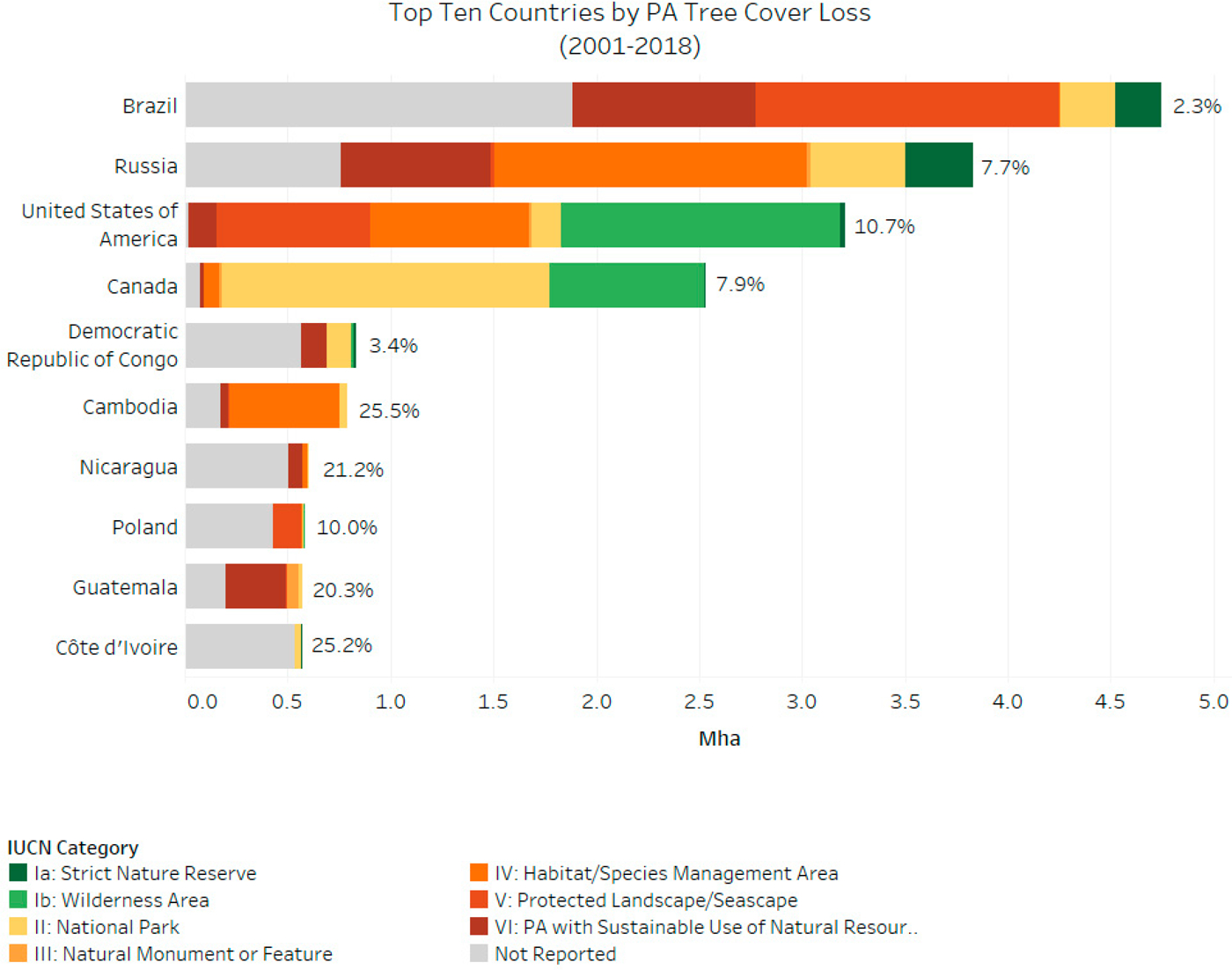
Top 10 countries with tree cover loss in PAs, by Union for Conservation of Nature (IUCN) category from 2001–2018. Bars represent tree cover loss in Mha, percentage is proportion of total PA tree cover lost in each country between 2001–2018. (See Supplementary Table S4 for full list of country-level results).

**Figure 4.**
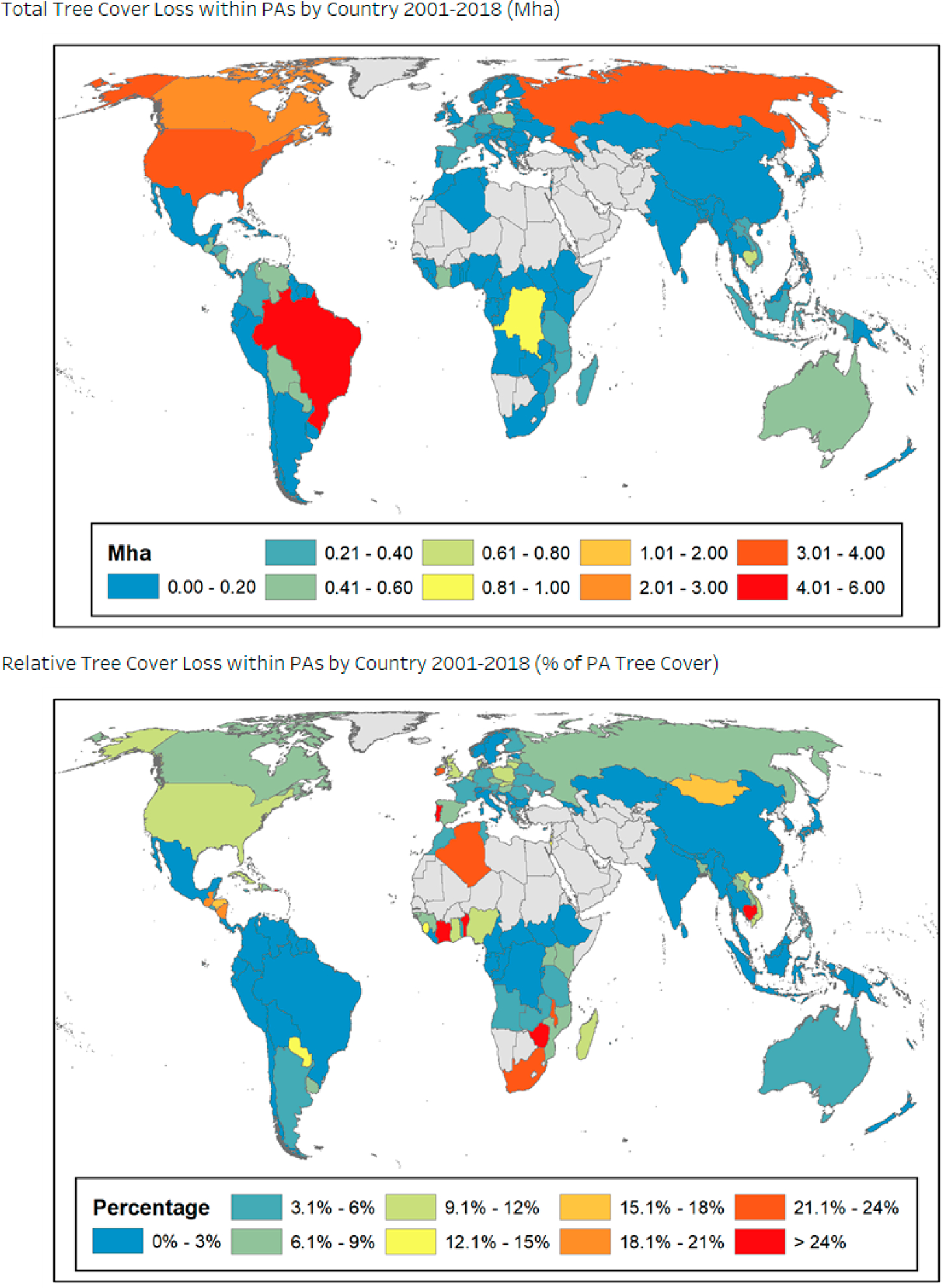
Top: total tree cover loss in PAs by country from 2001–2018 (Mha). Bottom: percentage of tree cover lost within PAs from 2001–2018.

South and Central America are responsible for the largest proportion, 32%, of forest loss in PAs over the study period, followed by North America (20%), Eastern Europe (18%), and Africa and the Middle East (12%). Forest loss in PAs increased across several regions over the study period, including Eastern Europe, Southeast Asia, Africa, and the Middle East, and in particular, in South and Central America (Figure 3). Specifically, Brazil is found to be the largest contributor to this increase in tree cover loss over time, with exceptionally high amounts of tree cover loss in 2016–2017. We also find that no regions experienced a substantial decline in forest loss in PAs over 2001–2018. Total and proportional forest loss by country is shown in Figure 4 (omitting countries with less than 1000 ha of tree cover in PAs) and presented in Supplementary Table S4.

**Figure 5.**
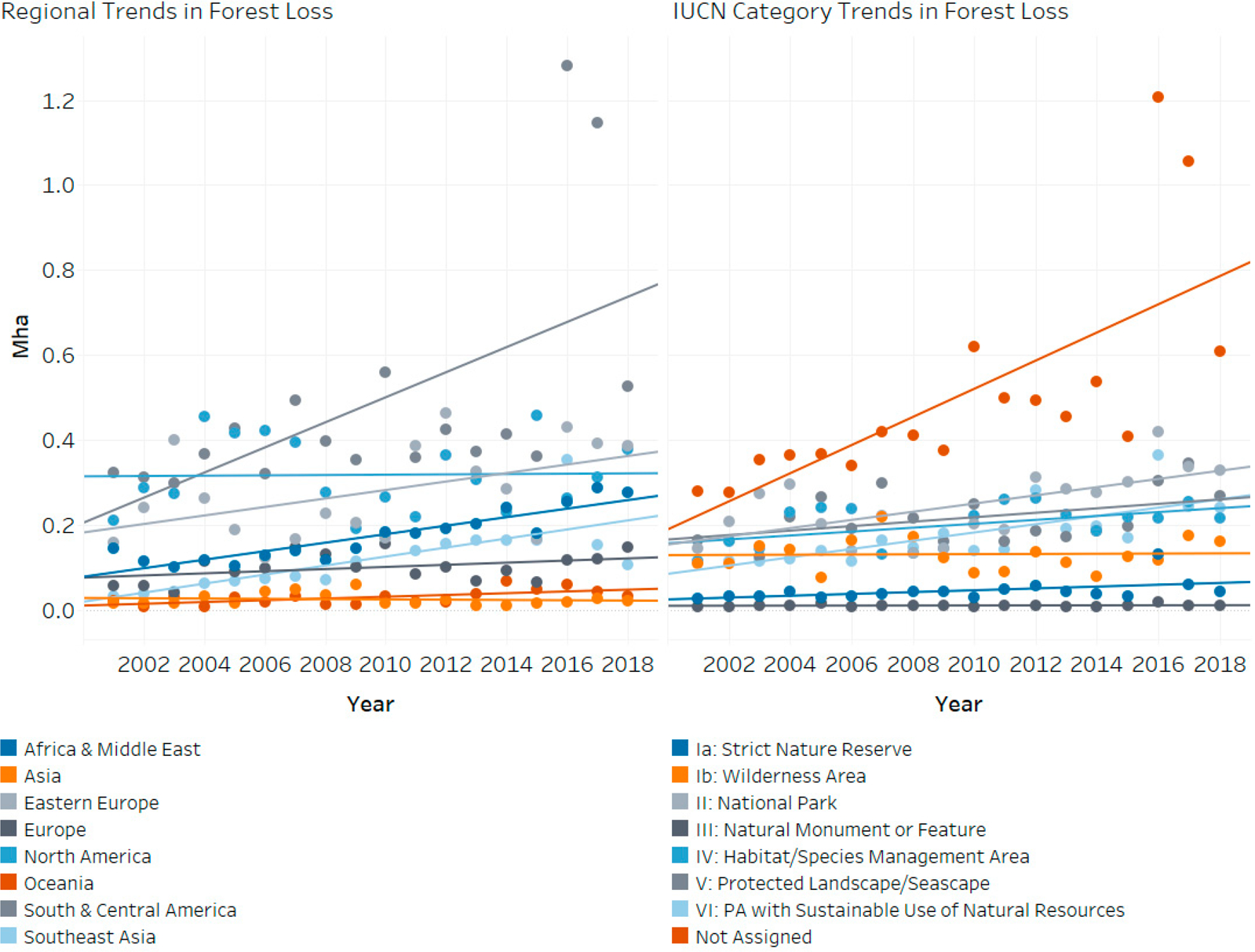
Left: historical trend in tree cover loss in PAs by region. Right: historical trend in tree cover loss by IUCN Category.

Forest loss by IUCN PA classification follows expected trends, with stricter categories of PA experiencing less loss than categories which allow some form of sustainable use (Figure 5, which shows annual PA tree cover loss by country (left) and by IUCN Category (right) with trendlines shown for reference). Categories Ia (Strict nature reserve), Ib (Wilderness Area), and III (National Monuments) have low annual forest loss and no noticeable trend over time. On the other hand, less strict categories, including IV (Habitat and Species Management Areas) and VI (Protected area with sustainable use of natural resources), experienced higher, and somewhat increasing, forest loss during the period between 2001 and 2018. However, PAs which do not fit into the IUCN classification scheme have both the highest amounts of tree cover loss, and the highest rate of increase in tree cover loss over time, but without more detailed management information, we cannot determine whether this is sanctioned clearing. Concerningly, National Parks (category II) should also be very strictly protected, but forest loss in these PAs doubled over the 2001–2018 period.

### 3.2. Land Cover Following Forest Loss in Protected Areas

**Figure 6.**
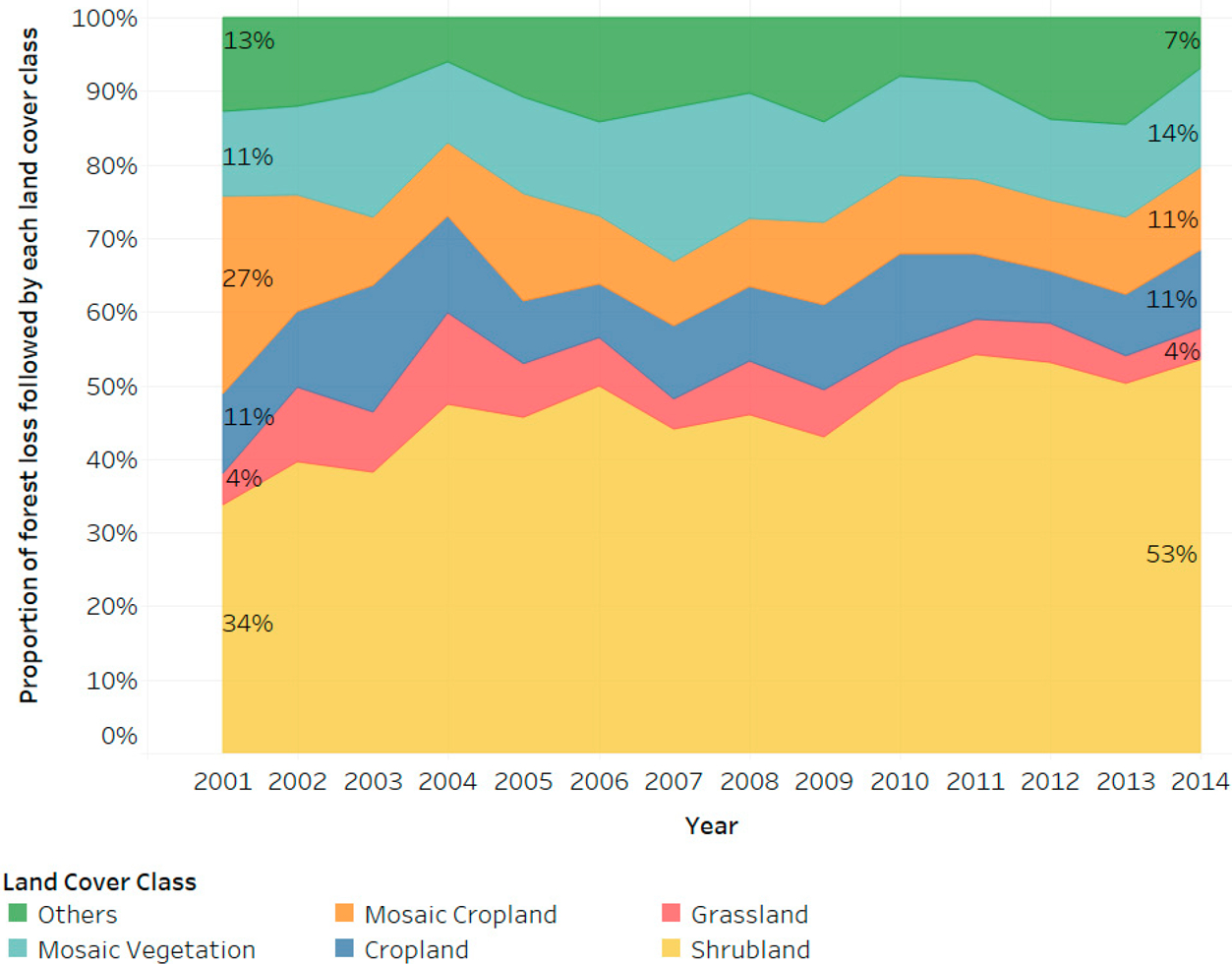
The proportion of forest loss within PAs followed by each land cover category, globally from 2001 to 2018, with the first and last year proportion labeled for reference.

Globally, across all PAs, shrubland is the dominant land cover following forest loss over 2001–2014, comprising almost half (47%) of all observations. Shrublands comprise a broad land cover category that could include re-growing forest, agricultural fallows, or pasture lands in some regional contexts. The proportion of forest loss in PAs followed by agriculture, including both cropland and mosaic cropland, is 22% (Figure 6). Another 14% of forest loss in PAs is followed by mosaic vegetation (which has the potential to be interspersed with small scale agriculture), and 6% by grassland. The remaining 11% of forest loss is followed by ‘other’ land uses including urban areas, water bodies, and bare areas. The proportion of forest loss followed by shrubland is the only category that significantly increased over 2001–2014, from about 35% in 2001 to more than 50% in 2014. On the other hand, mosaic cropland and grassland categories have decreased over the study period (Figure 6).

**Figure 7.**
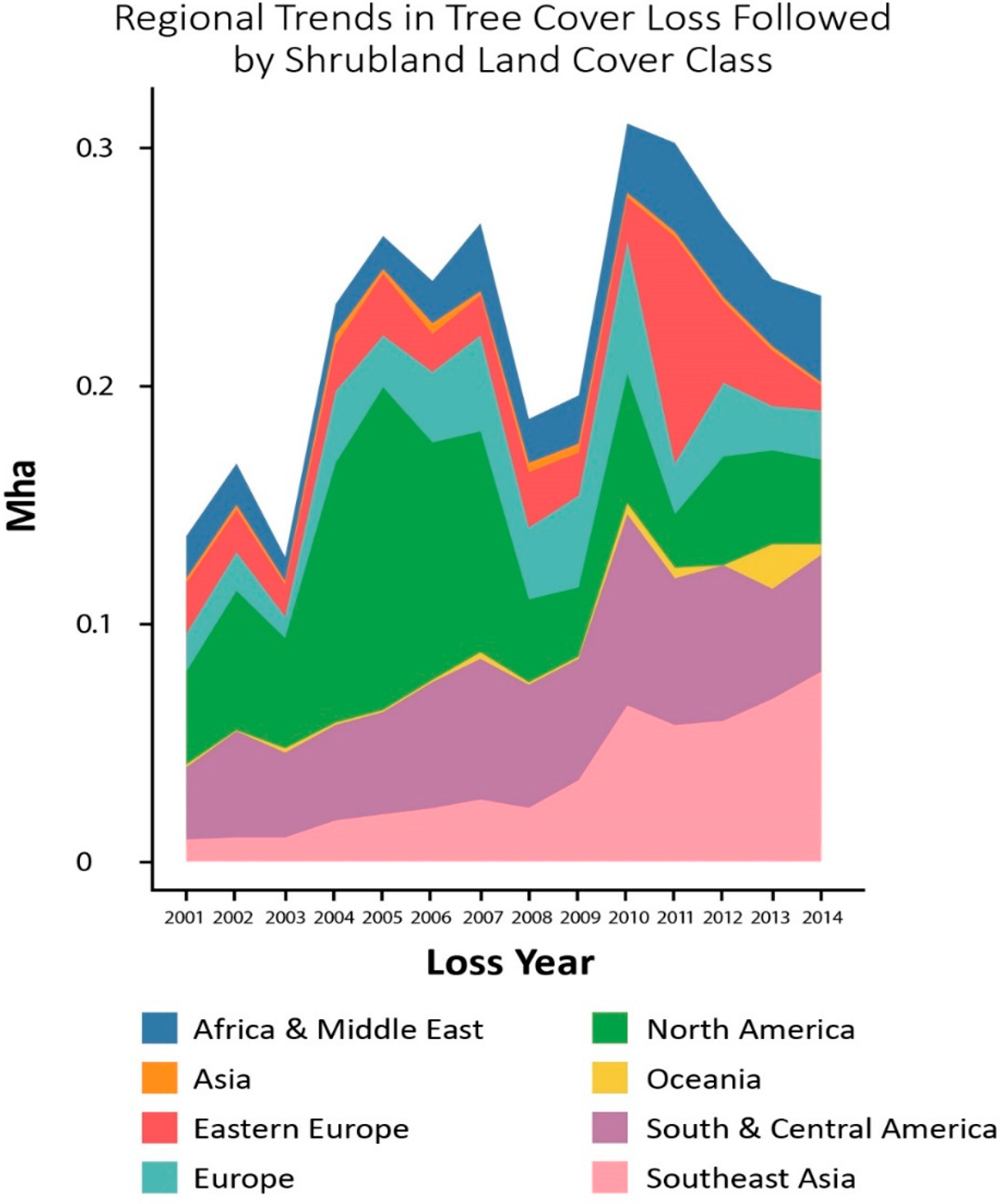
Calculated regional distribution of tree cover loss followed by shrubland from 2001 to 2014 (Mha).

Additionally, PA tree cover loss varies by regions (Figure 7). Early in the study period, South and Central America, Africa and the Middle East, and North America experienced the greatest proportion of tree cover loss followed by shrubland (42%, 19%, and 18% of global total, respectively) (shown in Figure 7). In later periods, the share of tree cover loss followed by shrubland declined in South and Central America, and North America (to 28% and 12%, respectively), while it continued to increase in Africa and the Middle East and Southeast Asia (from 19% in 2001 to 23% in 2014, and from 5% in 2001 to 23% in 2014, respectively).

### 3.3. Case Studies

We identified four case studies to illustrate varying drivers of land conversion, based on those countries and PAs with significant tree cover loss. For these PAs, in Brazil, Indonesia, Democratic Republic of Congo, and the United States, we examined high spatial resolution orthoimages to develop a more detailed understanding of the land cover following loss in these cases. This is not intended as a systematic validation of our analysis, but rather an illustration of how the global analysis can be followed by more detailed investigation with higher resolution satellite imagery.

Brazil’s Triunfo do Xingu Environmental PA (IUCN Category V) has recently been noted as a hotspot of deforestation due to pasture expansion, with more than 14,000 hectares of protected land converted to pasture over a six month period in 2018, and over 350,000 ha converted since 2006 [24]. Our analysis found that between 2001 to 2018, over 560,000 ha had experienced tree cover loss. Based on our global analysis, almost 40% of loss in this PA is followed by shrubland and grassland, and another 40% by mosaic agriculture from 2001 to 2015. Using high-resolution imagery from Google Earth, we observed that the forest loss in this PA appears to be organized along roads and settlements and in rectilinear configurations characteristic of agriculture and pastureland, with substantial grassland cover. This configuration suggests that indeed much of the grassland and shrubland cover following loss is managed for livestock grazing and emphasizes the challenge of distinguishing managed and unmanaged grasslands [22,25].

**Figure 8.**
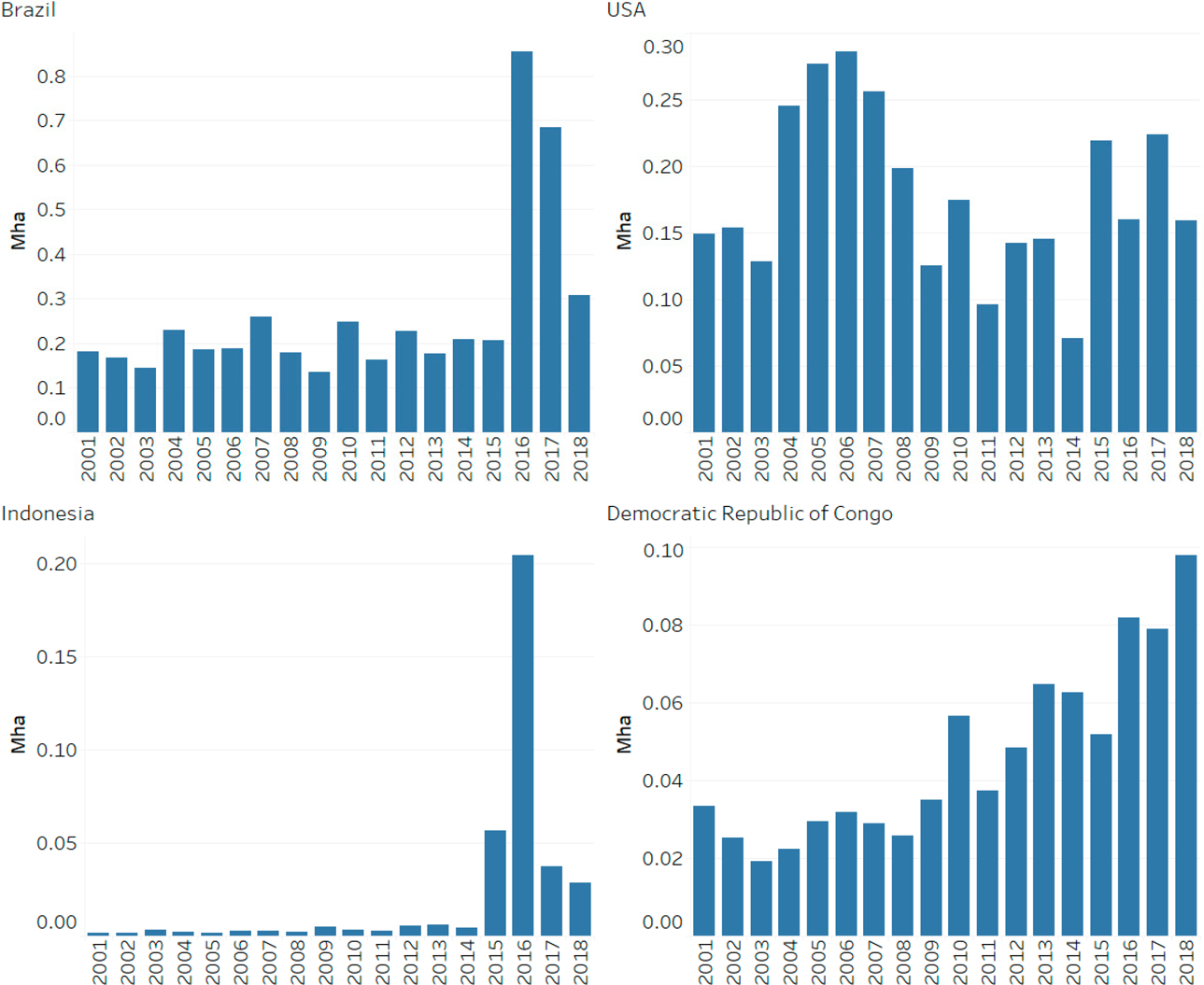
Historical trends in tree cover loss within PAs across selected countries from 2001 to 2018, note the y-axis is not consistent across each graph (Mha).

In 2016, just two PAs hosted 40% of forest loss in Indonesian PAs: Tanjung Puting National Park (IUCN Category II) had tree cover loss of about 470 km^2^ of 3250 km^2^ of total tree cover, and Sebangau National Park (IUCN Category II) which had tree cover loss of about 460 km^2^ of 5700 km^2^ of total tree cover, both peat forest PAs in the Central Kalimantan region. As the majority of this loss occurred after 2015, we do not have results from our global analysis about the subsequent land cover. However, more detailed examination of loss patterns in Sebangau National Park (Figure 8) suggests that forest loss is generally followed by grassland or shrubland. This largely conforms to findings from previous research highlighting the important role of fires, generally anthropogenic in origin but unintentionally impacting large expanses of peat forests, in driving deforestation across Central Kalimantan since 2015 [15].

Democratic Republic of Congo has the fifth highest rate of forest loss in PAs and experienced a steadily increasing rate of forest loss in PAs over 2001–2018. The majority of this loss occurred in PAs categories without an IUCN category (“Not applicable”). We examined loss in the Sankuru Nature Reserve, which was created in 2007 to protect Bonobo habitat and is managed by local communities [26]. Our global analysis found that more than 90% of forest loss in Sankuru was followed by crop land, including mosaic agriculture (in total we found that 1100 km^2^ of 26,700 km^2^ of tree cover was lost). Our detailed examination of imagery on google earth confirmed that the majority of the land cover in areas of loss was small-scale agriculture along roads and near urban areas.

**Figure 9.**
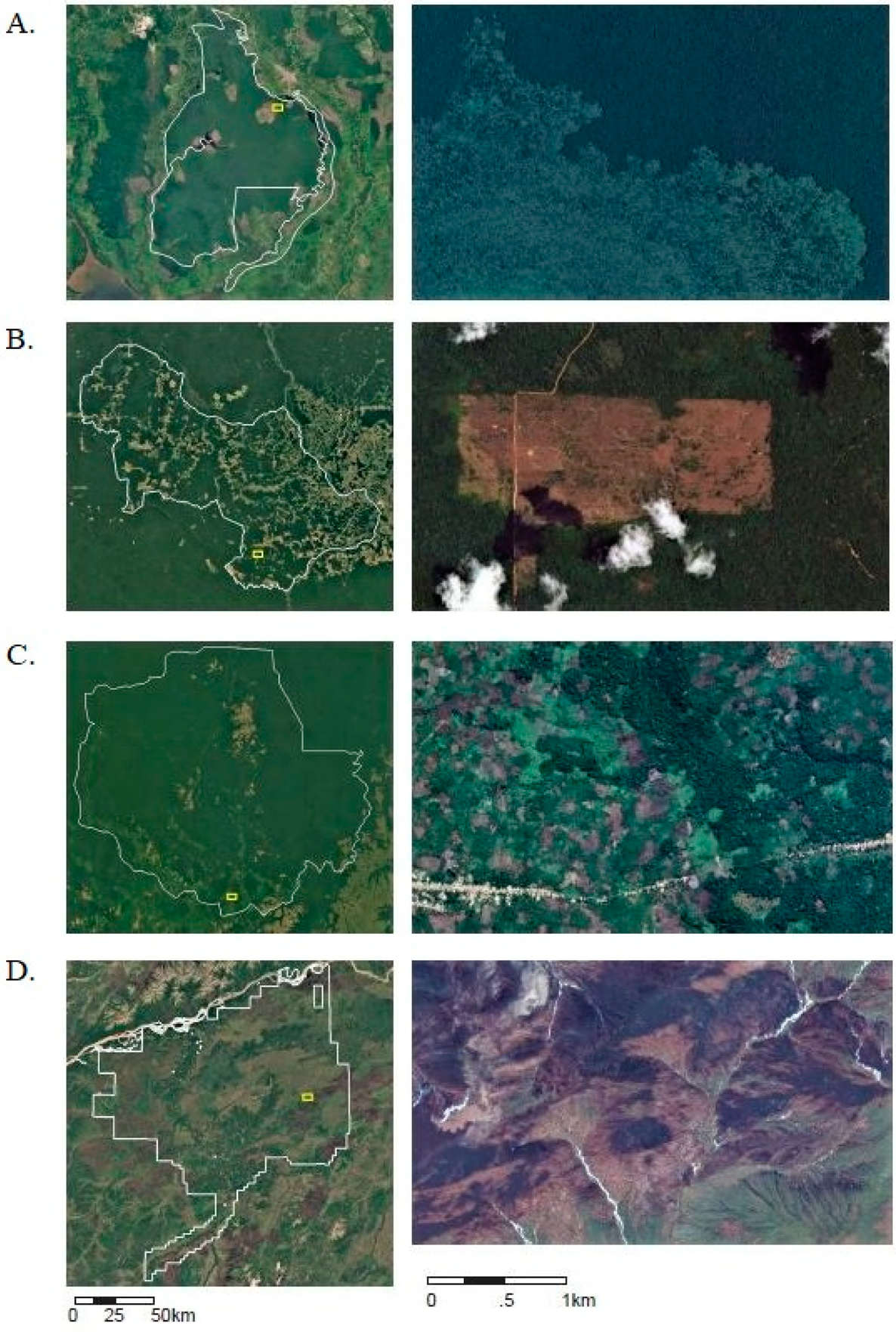
Selected PA imagery showcasing deforestation events across case studies: (**A**) Sebangau National Park in Indonesia, (**B**) Triunfo do Xingu Environmental PA in Brazil, (**C**) Sankuru Nature Reserve in Democratic Republic of Congo, and (**D**) Nowitna National Wildlife Refuge in the United States. Images were collected from Google Earth and represent the years 2018 or 2019. Zoomed out images on the left are from the United States Geological Survey (USGS) and the National Aeronautics and Space Administration’s (NASA) Landsat program, while zoomed in images on the right are satellite imagery products from Digital Globe.

Between 2001–2018, 11.4% of global forest loss in PAs occurred in the US, where loss remained relatively stable over time (Figure 8). We examined forest loss trends in Nowitna National Wildlife Refuge (IUCN Category IV) in Alaska, which regularly experiences wildfires and associated forest loss. Between 2001 to 2015, we found that out of about 5700 km^2^ of forest cover within this PA, more than 1200 km^2^ of forest cover was lost. Our global analysis found that nearly all the forest loss in this PA was followed by shrubland and grassland (99.6%). High-resolution imagery suggests that forest loss in Nowitna does appear to be caused by wildfires, which leave burn scars and are followed by a mosaic vegetation dominated by shrubland and grassland categories (Figure 9).

## 4. Discussion

Between 2001 and 2018, two trends took place simultaneously. The absolute area of protected tree cover increased due to countries designating additional land as PAs. Conversely, the annual rate of tree cover loss inside PAs nearly doubled during this time period. The highest loss in tree cover within PAs occurred in 2016, when 0.44% of protected forests experienced loss. Globally, it seems that forests in PAs face the same economic, natural, and social pressures as non-protected forests, as shown by the consistency in trends of forest loss between the two forests categories (Figure 2). This is despite PAs being in areas which should experience fewer human pressures of deforestation [9,11].

In the tropics, the extent of forest loss in PAs increased notably over the study period, and occurred largely in Indonesia, Brazil, and the Democratic Republic of Congo. Global land cover maps demonstrate that shrublands and grasslands were the dominant land cover following forest loss in PAs in the tropics. Our case study analysis demonstrated that this loss corresponds to areas impacted by fires, for example in Indonesian peat lands, and may also correspond to pasture land, for example in Brazil. In many countries in the tropics, agriculture was also a dominant land cover following forest loss, particularly in Sub-Saharan Africa.

In the northern hemisphere, the United States, Canada, and Russia contribute large total amounts of protected tree cover loss. Unlike in the tropics, forest loss in PAs in these countries did not increase noticeably over time. But similarly, shrublands were also the dominant land cover following forest loss over the study period. It is likely that most of this loss corresponds to natural occurrences such as fire, pests, or storm blowdown, which have been shown to be dominant drivers of deforestation in these regions outside of PAs [17]. Indeed, our US case study identified wildfire as a dominant driver of loss.

PAs that do not fit within IUCN categorization schema, which comprise roughly one-third of all PAs, have the highest rates of tree cover loss. These PAs include indigenous lands and UNESCO reserves. There is evidence that indigenous land tenure recognition is effective at preventing deforestation [27]. On the other hand, the majority of uncategorized PAs do not have any active management authority. It is possible that a lack of clear authority over these PAs may be one reason for higher rates of tree cover loss in these uncategorized PAs as a whole.

An important limitation of our assessment of land cover following forest loss in PAs is the reliance on a global mid-resolution land cover dataset. We used a global approach to allow for direct comparisons across regions, and to identify specific regions (within and across countries) where further investigation is needed. However, global land cover datasets do not necessarily address land use and may struggle, for example, to differentiate grazing and pasture lands from shrublands or grasslands [22]. This limits our ability to reliably track pasture expansion into PAs in some geographies where it is important, including in Brazil. Also, mosaic land cover classes such as shrubland/mosaic natural vegetation according to the global map could actually be agroforestry or mixed cropland/agroforestry. This limits our ability to track small-scale and mixed agriculture classes in geographies where those are dominant land cover transitions, such as Central Africa.

We used a case study approach to gauge availability and usefulness of the additional information available via high-resolution imagery from Google Earth to identify and track drivers of forest loss in PAs. This was not intended as a validation of the global approach, but rather an exploration of the potential utility of this emerging technology for more in-depth examination of drivers of forest loss in hotspots of deforestation or priority conservation areas. We found that the spatial and temporal resolution of the imagery available on Google Earth helped inform possible reasons for forest loss, including wildfire, small-scale agriculture, and pasturelands.

Finally, the ESA-CCI dataset represents land cover at 300 m resolution, so pixels with a small proportion of a given land cover category may not be represented, even if they are identified in the 30 m resolution tree cover loss dataset (also discussed in Reference [20]). This will impact our results in areas with highly heterogenous land cover, or small and isolated deforestation events such as targeted logging operations. Recent studies report that logging is the most common driver of loss in intact, but not necessarily protected, forests globally [28]. Logging is difficult to detect via satellite imagery because in many cases, sufficient canopy cover remains following logging that land cover is still classified as forest. High-spatial resolution and frequent satellite imagery may be able to detect the most evident indications of logging, including access roads, skid trails, and tree fall gaps. However, research suggests that these may comprise as little as 20% of the total area impacted by logging activities [29]. Because we use a relatively coarse resolution land cover map, very small-scale or ephemeral forest disturbances—even isolated tree cover loss events in the GFC loss map—will be reported as followed by forest cover. Future research with higher resolution imagery could support investigation of the role of logging in PAs globally.

Despite limitations, by calculating tree cover loss at the PA level, we now have a comprehensive global dataset that can be used to compare outcomes across PAs, to identify the specific characteristics of PAs which limit the rate of tree cover loss over time, and to evaluate the impact of PAs on reducing tree cover loss. There is a growing literature aimed at measuring the impact of human pressure on PAs [3]. This dataset can complement future studies which aim to assess the impacts of socio- and macro-economic factors on ecosystem degradation within PAs. Also, as the land use sector is increasingly recognized for its important role in stabilizing future climate, this research can inform assumptions of land available for agriculture. Given that agriculture is occurring in PAs in some regions, despite their designation, researchers and modelers may not want to assume that all protected land will remain in a natural state to more accurately represent land cover dynamics globally.

## 5. Conclusions

Though PAs are a key strategy for safeguarding global biodiversity and ecosystem services, they remain under threat from a range of direct and indirect drivers of forest loss [2–4,6]. We found that between 2001 and 2018, global PAs lost 25.5 Mha of forest, or 4.1% of their forested area. This study aimed to improve our understanding of why this loss occurred by examining the land cover following forest loss in PAs. We found that shrubland was the dominant land cover following forest loss in PAs and became increasingly dominant over the study period. This may reflect the fact that the shrubland category encompasses a range of land cover types and land uses, including burned and regenerating forests, fallow lands, and possibly pasture lands, that have been shown to have extensive impacts in key deforestation hotpots globally [15–17]. Agriculture was not the most prominent land cover following forest loss events in PAs globally, but agriculture was shown to be prominent in key geographies—many in Sub-Saharan Africa, including Nigeria, Ghana, and Côte d’Ivoire (Supplementary Table S4). Our analysis improves our understanding of the causes of forest loss in PAs globally and at regional/national scales and can be used to broadly inform strategies to improve PA management and enforcement that are tailored to these agents of change.

## Supplementary Material

Supplement

## Figures and Tables

**Table 1.** Summary of spatial datasets used.

Parameter	Years Represented	Spatial Resolution	Reference	Version
Protected Areas (WDPA^1^)	2017	Vector	[5]	1.5
Tree Cover Loss (GFC^2^)	Annual 2001–2018	30 m	[18]	1.6
Land Cover (ESA-CCI^3^)	2005, 2010, 2015	300 m	[19]	2.0.7

1. World Database on Protected Areas

2. Global Forest Change

3. European Space Agency-Climate Change Initiative

## References

[1] UNEP-WCMC (2018). User Manuel for the World Database on Protected Areas and world database on other effective area-based conservation measures: 1.5 UNEP-WCMC: Cambridge, UK Available at http://wcmc.io/WDPA_Manual

[2] Lindsey PA; Nyirenda VR; Barnes JI; Becker MS; McRobb R; Tambling CJ; Taylor WA; Watson FG; t’Sas-Rolfes M Underperformance of African protected area networks and the case for new conservation models: insights from Zambia. PLoS one 2014, 9, e94109. 24847712 10.1371/journal.pone.0094109 PMC4029602

[3] Jones KR; Venter O; Fuller RA; Allan JR; Maxwell SL; Negret PJ; Watson JE One-third of global protected land is under intense human pressure. Science 2018, 360, 788–791. 29773750 10.1126/science.aap9565

[4] Geldmann J; Joppa LN; Burgess ND Mapping change in human pressure globally on land and within protected areas. Conservation Biology 2014, 28, 1604–1616. 25052712 10.1111/cobi.12332

[5] UNEP-WCMC. User Manual for the World Database on Protected Areas and world database on other effective area-based conservation measures: 1.6 UNEP-WCMC: Cambridge, UK, 2019.

[6] Heino M; Kummu M; Makkonen M; Mulligan M; Verburg PH; Jalava M; Räsänen TA Forest loss in protected areas and intact forest landscapes: a global analysis. PLoS One 2015, 10, e0138918. 26466348 10.1371/journal.pone.0138918 PMC4605629

[7] Pressey R; Whish G; Barrett T; Watts M Effectiveness of protected areas in north-eastern New South Wales: recent trends in six measures. Biological Conservation 2002, 106, 57–69.

[8] Andam KS; Ferraro PJ; Pfaff A; Sanchez-Azofeifa GA; Robalino JA Measuring the effectiveness of protected area networks in reducing deforestation. Proceedings of the national academy of sciences 2008, 105, 16089–16094. 10.1073/pnas.0800437105 PMC2567237 18854414

[9] Joppa LN; Pfaff A High and far: biases in the location of protected areas. PloS one 2009, 4, e8273. 20011603 10.1371/journal.pone.0008273 PMC2788247

[10] Jones KW; Lewis DJ Estimating the counterfactual impact of conservation programs on land cover outcomes: The role of matching and panel regression techniques. PloS one 2015, 10, e0141380. 26501964 10.1371/journal.pone.0141380 PMC4621053

[11] Venter O; Magrach A; Outram N; Klein CJ; Possingham HP; Di Marco M; Watson JE Bias in protected‐area location and its effects on long‐term aspirations of biodiversity conventions. Conservation Biology 2018, 32, 127–134. 28639356 10.1111/cobi.12970

[12] Burivalova ZA; Allnutt FT; Rademacher D; Schlemm A; Wilcove DS; Butler RA What works in tropical forest conservation, and what does not: Effectiveness of four strategies in terms of environmental, social, and economic outcomes. Conservation Science and Practice 2019, 1, doi: 10.1111/csp2.28.

[13] Bebber DP; Butt N Tropical protected areas reduced deforestation carbon emissions by one third from 2000–2012. Scientific reports 2017, 7, 14005. 29070820 10.1038/s41598-017-14467-w PMC5656627

[14] Brun C; Cook AR; Lee JSH; Wich SA; Koh LP; Carrasco LR Analysis of deforestation and protected area effectiveness in Indonesia: A comparison of Bayesian spatial models. Global Environmental Change 2015, 31, 285–295, doi:10.1016/j.gloenvcha.2015.02.004.

[15] Austin KG; Schwantes A; Gu Y; Kasibhatla PS What causes deforestation in Indonesia? Environmental Research Letters 2019, 14, 024007.

[16] De Sy V; Herold M; Achard F; Beuchle R; Clevers J; Lindquist E; Verchot L Land use patterns and related carbon losses following deforestation in South America. Environmental Research Letters 2015, 10, 124004.

[17] Curtis PG; Slay CM; Harris NL; Tyukavina A; Hansen MC Classifying drivers of global forest loss. Science 2018, 361, 1108–1111. 30213911 10.1126/science.aau3445

[18] Hansen MC; Potapov PV; Moore R; Hancher M; Turubanova S; Tyukavina A; Thau D; Stehman S; Goetz S; Loveland TR High-resolution global maps of 21st-century forest cover change. science 2013, 342, 850–853. 24233722 10.1126/science.1244693

[19] ESA. Land Cover CCI Product User Guide Version 2. Tech. Rep 2017 Available at: maps.elie.ucl.ac.be/CCI/viewer/download/ESACCI-LC-Ph2-PUGv2_2.0.pdf

[20] Pendrill F; Persson UM Combining global land cover datasets to quantify agricultural expansion into forests in Latin America: Limitations and challenges. PloS one 2017, 12, e0181202. 28704510 10.1371/journal.pone.0181202 PMC5509295

[21] Initiative, E.S.A.C.C. Land Cover State Products in GTiff format at 300-meter resolution. ESA-CCI, Ed. 2015.

[22] Ali I; Cawkwell F; Dwyer E; Barrett B; Green S Satellite remote sensing of grasslands: from observation to management. Journal of Plant Ecology 2016, 9, 649–671, doi:10.1093/jpe/rtw005.

[23] Weisse M; Goldman L Global Tree cover loss rose 51 percent in 2016. World Resources Institute. Available online at: http://www.wri.org/blog/2017/10/global-tree-cover-loss-rose-51-percent-2016 (accessed on September 2019).

[24] Gaworecki M Pasture expansion driving deforestation in Brazilian protected area. Mongabay Series. Available online at: https://news.mongabay.com/2018/10/pasture-expansion-driving-deforestation-in-brazilian-protected-area/ (Accessed September 2019).

[25] Ogle SM; McCarl BA; Baker J; Del Grosso SJ; Adler PR; Paustian K; Parton WJ Managing the nitrogen cycle to reduce greenhouse gas emissions from crop production and biofuel expansion. Mitigation and adaptation strategies for global change 2016, 21, 1197–1212.

[26] Volckhausen T Bonobo conservation stymied by deforestation, human rights abuses. Mongabay. Available online at: https://news.mongabay.com/2019/10/bonobo-conservation-stymied-by-deforestation-human-rights-abuses/ (accessed 30 October 2019).

[27] Busch J; Ferretti-Gallon K What Drives Deforestation and What Stops It? A Meta-Analysis. Review of Environmental Economics and Policy 2017, 11, 3–23, doi:10.1093/reep/rew013.

[28] Scullion JJ; Vogt KA; Drahota B; Winkler-Schor S; Lyons M Conserving the Last Great Forests. A Meta-Analysis Review of the Drivers of Intact Forest Loss and the Policies and Strategies to Save Them. Frontiers in Forests and Global Change 2019, 2, 62.

[29] Pearson TR; Bernal B; Hagen SC; Walker SM; Melendy LK; Delgado G Remote assessment of extracted volumes and greenhouse gases from tropical timber harvest. Environmental Research Letters 2018, 13, 065010.

