# Supplementary material for: What is Threatening Forests in Protected Areas? A Global Assessment of Deforestation in Protected Areas, 2001–2018
Source: Forests. Author manuscript; Available in PMC 2020 Oct 28. (PMC7592705; doi:10.3390/f11050539)
Supplement: Supplement [file NIHMS1631712-supplement-Supplement.docx]

Supplement


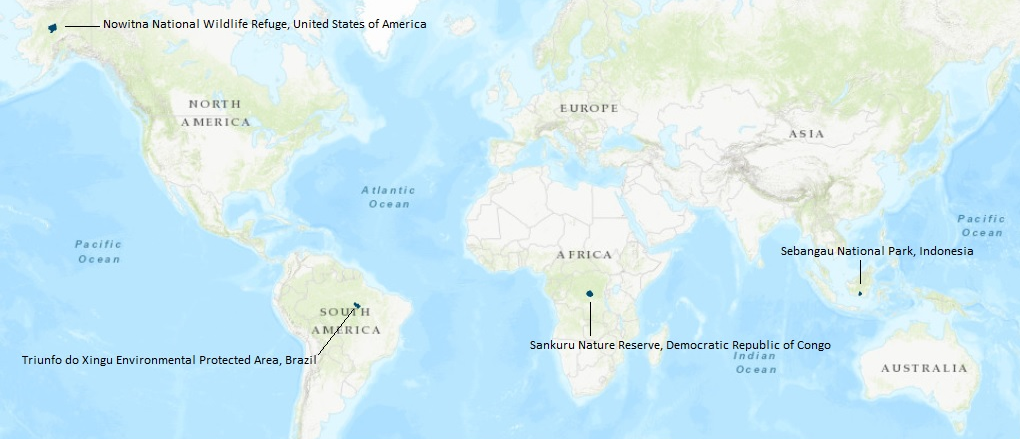


**Figure S1.** Location of case studies.

**Table S1.** Definitions and key features of PA types, adopted from IUCN.

| Classification | Description | Key Features |
| --- | --- | --- |
| Ia Strict Nature Reserve | Protected areas that are strictly set aside to protect biodiversity and also possibly geological/geomorphological features, where human visitation, use and impacts are strictly controlled and limited to ensure protection of the conservation values. Such protected areas can serve as indispensable reference areas for scientific research and monitoring. | Largely complete set of expected native species in ecologically significant densities; have a full set of native ecosystems; largely intact; be free of direct intervention by modern humans; not require substantial interventions |
| Ib Wilderness Area | Protected areas that are usually large unmodified or slightly modified areas, retaining their natural character and influence, without permanent or significant human habitation, which are protected and managed so as to preserve their natural condition. | Be free of modern infrastructure, and industrial extractive activity; be characterized by a high degree of intactness; be of sufficient size to protect biodiversity, maintain ecological processes and ecosystem services; be free of inappropriate or excessive human use or presence |
| II National Park | Large natural or near natural areas set aside to protect large-scale ecological processes, along with the complement of species and ecosystems characteristic of the area, which also provide a foundation for environmentally and culturally compatible spiritual, scientific, educational, recreational and visitor opportunities. | Typically, large and conserve a functioning ecosystem, but may need to be complemented by sympathetic management is some areas; should contain representative examples of major natural regions, and biological and environmental features or scenery; should keep composition, structure, and function of biodiversity in a “natural” state |
| III Natural Monument or Feature | Protected areas set aside to protect a specific natural monument, which can be a landform, sea mount, submarine cavern, geological feature such as a cave or even a living feature such as an ancient grove. They are generally quite small protected areas and often have high visitor value. | Usually relatively small sites that focus on one or more prominent natural features and the associated ecology; includes natural geological and geomorphological features; culturally-influenced natural features; natural-cultural sites |
| IV Habitat/Species Management Area | Protected areas aiming to protect particular species or habitats and management reflects this priority. Many category IV protected areas will need regular, active interventions to address the requirements of particular species or to maintain habitats, but this is not a requirement of the category. | Aim to protect or restore: flora species, fauna species, and/or habitats of international, national, or local importance. Most contain actitive management to maintain target species, natural habitats, or culturally-defined ecosystems |
| V Protected Landscape/Seascape | A protected area where the interaction of people and nature over time has produced an area of distinct character with significant ecological, biological, cultural and scenic value: and where safeguarding the integrity of this interaction is vital to protecting and sustaining the area and its associated nature conservation and other values. | Result from biotic, abiotic, and human interaction. Its essential for Category V protected areas to include landscape and/or coastal and island seascape of high and/or distinct scenic quality and with significant associated habitats, flora and fauna and associated cultural features; include a balanced interaction between people and nature; unique or traditional land-use patterns |
| VI Protected area with sustainable use of natural resources | Protected areas that conserve ecosystems and habitats, together with associated cultural values and traditional natural resource management systems. They are generally large, with most of the area in a natural condition, where a proportion is under sustainable natural resource management and where low-level non-industrial use of natural resources compatible with nature conservation is seen as one of the main aims of the area. | Aim to have the sustainable use of natural resources as a means to achieve nature conservation, together and in synergy with other actions such as protection; not designed to accommodate large-scale industrial harvest |

**Table S2.** Reclassification schema for ESA-CCI land cover dataset.

| Original Code | Land Cover Description | Reclass Code | Reclass Description |
| --- | --- | --- | --- |
| 10 | Cropland, rainfed | 1 | Cropland |
| 11 | Herbaceous cover | 5 | Shrubland |
| 12 | Tree or shrub cover | 5 | Shrubland |
| 20 | Cropland irrigated or post-flooding | 1 | Cropland |
| 30 | Mosaic cropland (crop land >50% / tree, shrub, herbaceous cover <50%) | 2 | Mosaic cropland |
| 40 | Mosaic natural vegetation (tree, shrub, herbaceous cover >50% / cropland <50%) | 3 | Mosaic vegetation |
| 50 | Tree cover, broadleaved, evergreen, closed to open (>15%) | 4 | Forest |
| 60 | Tree cover, broadleaved, deciduous, closed to open (>15%) | 4 | Forest |
| 61 | Tree cover, broadleaved, deciduous, closed (>40%) | 4 | Forest |
| 62 | Tree cover, broadleaved, deciduous, open (15-40%) | 4 | Forest |
| 70 | Tree cover, needle-leaved, evergreen, closed to open (>15%) | 4 | Forest |
| 71 | Tree cover, needle-leaved, evergreen, closed (>40%) | 4 | Forest |
| 72 | Tree cover, needle-leaved, evergreen, open (15-40%) | 4 | Forest |
| 80 | Tree cover, needle-leaved, deciduous, closed to open (>15%) | 4 | Forest |
| 81 | Tree cover, needle-leaved, deciduous, closed (>40%) | 4 | Forest |
| 82 | Tree cover, needle-leaved, deciduous, open (15-40%) | 4 | Forest |
| 90 | Tree cover, mixed leaf type (broadleaved and needle-leaved) | 4 | Forest |
| 100 | Mosaic Tree and shrub (>50%) / herbaceous cover (<50%) | 5 | Shrubland |
| 110 | Mosaic herbaceous cover (>50%) / T and shrub (<50%) | 5 | Shrubland |
| 120 | Shrubland | 5 | Shrubland |
| 121 | Shrubland evergreen | 5 | Shrubland |
| 122 | Shrubland deciduous | 5 | Shrubland |
| 130 | Grassland | 6 | Grassland |
| 140 | Lichens and mosses | 7 | Other |
| 150 | Sparse vegetation (tree, shrub, herbaceous cover) (<15%) | 5 | Shrubland |
| 151 | Sparse tree (<15%) | 5 | Shrubland |
| 152 | Sparse shrub (<15%) | 5 | Shrubland |
| 153 | sparse herbaceous cover (<15%) | 5 | Shrubland |
| 160 | Tree cover, fresh or brackish water | 4 | Forest |
| 170 | Tree cover, flooded, saline water | 4 | Forest |
| 180 | Shrub or herbaceous cover, flooded, fresh/saline/brackish water | 5 | Shrubland |
| 190 | Urban areas | 7 | Other |
| 200 | Bare areas | 7 | Other |
| 201 | consolidated bare areas | 7 | Other |
| 202 | Unconsolidated bare areas | 7 | Other |
| 210 | Water bodies | 7 | Other |
| 220 | Permanent snow and ice | 7 | Other |

**Table S3.** Comparison of land cover following deforestation results from this study and previous studies [15, 16]. Shown as percent of total deforested area.

| Study Region | Study | Agriculture^1^ | Forestry | Grassland/Shrubland | Other |
| --- | --- | --- | --- | --- | --- |
| **Indonesia (national)** | **Austin (2019)** | 67.0% | 9.3% | 19.7% | 4.0% |
|  | **This Study** | 60.7% | 30.0% | 9.0% | 0.3% |
| **Indonesia (PAs)** | **Austin (2019)** | 40.4% | 32.4% | 18.3% | 8.9% |
|  | **This Study** | 38.2% | 47.4% | 7.9% | 6.5% |
| **South America** | **De Sy (2015)** | 20% | Not included | 69% (pasture) | 11% |
|  | **This Study** | 44.2% | NA^2^ | 44.4% | 11.4% |

^1.^ Agriculture land classes include Industrial Cropland, Mosaic Cropland, and Mosaic Vegetation

^2.^ Deforested land returning to forestry was excluded for the purpose of comparison

**Table S4.** Total tree cover estimates (1000 ha), tree cover loss estimates from 2001-2018 (1000 ha), relative tree cover loss from 2001-2018 (%), tree cover loss followed by agricultural land type (industrial, mosaic, and total) from 2001-2014 (1000 ha), and proportion of total tree cover loss followed by agriculture (%) within PAs at the national level^[[1]](#footnote-1)^.

| Country | Name | PA Tree Cover 2018 | PA Tree Cover Loss 2001-2014 | PA Tree Cover Loss 2015-2018 | PA Tree Cover Loss 2001-2018 | % Tree Cover Loss in PAs 2001-2018 | PA Loss followed by Industrial Agriculture 2001-2014 | PA Loss followed by Mosaic Agriculture 2001-2014 | Total PA Loss followed by Agriculture 2001-2014 | % PA Loss followed by Ag 2001-2014 |
| --- | --- | --- | --- | --- | --- | --- | --- | --- | --- | --- |
| BRA | Brazil | 207,450 | 2697 | 2051 | 4749 | 2% | 25 | 208 | 232 | 9% |
| RUS | Russian Federation | 49,940 | 2793 | 1031 | 3825 | 8% | 15 | 17 | 33 | 1% |
| USA | United States of America | 29,890 | 2451 | 762 | 3214 | 11% | 25 | 17 | 42 | 2% |
| CAN | Canada | 32,130 | 1851 | 646 | 2497 | 8% | 1 | 37 | 38 | 2% |
| COD | Congo, Democratic Republic of the | 24,640 | 522 | 311 | 833 | 3% | 44 | 68 | 112 | 21% |
| KHM | Cambodia | 3,090 | 665 | 122 | 787 | 25% | 84 | 69 | 153 | 23% |
| NIC | Nicaragua | 2,820 | 330 | 267 | 597 | 21% | 1 | 2 | 3 | 1% |
| POL | Poland | 5,780 | 387 | 189 | 576 | 10% | 6 | 7 | 12 | 3% |
| GTM | Guatemala | 2,830 | 435 | 137 | 573 | 20% | 72 | 36 | 108 | 25% |
| CIV | Côte d'Ivoire | 2,250 | 427 | 142 | 569 | 25% | 117 | 63 | 180 | 42% |
| VEN | Venezuela (Bolivarian Republic of) | 31,410 | 359 | 194 | 553 | 2% | 31 | 28 | 59 | 16% |
| AUS | Australia | 9,940 | 314 | 170 | 483 | 5% | 13 | 6 | 19 | 6% |
| PRY | Paraguay | 3,080 | 335 | 115 | 450 | 15% | 2 | 14 | 15 | 5% |
| BOL | Bolivia (Plurinational State of) | 18,100 | 341 | 95 | 436 | 2% | 11 | 17 | 27 | 8% |
| IDN | Indonesia | 16,780 | 47 | 327 | 374 | 2% | 2 | 8 | 10 | 20% |
| VNM | Viet Nam | 3,010 | 264 | 100 | 364 | 12% | 8 | 19 | 28 | 11% |
| FRA | France | 5,710 | 285 | 77 | 362 | 6% | 8 | 6 | 15 | 5% |
| DEU | Germany | 6,770 | 290 | 64 | 355 | 5% | 7 | 5 | 11 | 4% |
| COL | Colombia | 13,890 | 188 | 85 | 273 | 2% | 5 | 7 | 12 | 6% |
| TZA | Tanzania, United Republic of | 4,510 | 210 | 53 | 263 | 6% | 8 | 20 | 28 | 13% |
| HND | Honduras | 1,490 | 137 | 107 | 244 | 16% | 2 | 0 | 3 | 2% |
| ESP | Spain | 3,540 | 166 | 77 | 243 | 7% | 4 | 3 | 7 | 4% |
| LAO | Lao People's Democratic Republic | 3,450 | 160 | 79 | 239 | 7% | 3 | 5 | 8 | 5% |
| MDG | Madagascar | 1,480 | 105 | 86 | 191 | 13% | 3 | 3 | 6 | 6% |
| MNG | Mongolia | 930 | 148 | 21 | 169 | 18% | 1 | 1 | 2 | 1% |
| ZMB | Zambia | 3,440 | 120 | 46 | 167 | 5% | 3 | 1 | 4 | 4% |
| PHL | Philippines | 3,060 | 75 | 76 | 152 | 5% | 9 | 19 | 27 | 36% |
| ZAF | South Africa | 660 | 113 | 37 | 149 | 23% | 3 | 3 | 6 | 5% |
| THA | Thailand | 7,700 | 110 | 32 | 143 | 2% | 12 | 8 | 19 | 18% |
| ARG | Argentina | 3,790 | 109 | 34 | 143 | 4% | 2 | 5 | 8 | 7% |
| MEX | Mexico | 6,730 | 101 | 40 | 141 | 2% | 4 | 3 | 8 | 8% |
| ROU | Romania | 2,890 | 108 | 31 | 139 | 5% | 2 | 1 | 3 | 3% |
| NGA | Nigeria | 1,400 | 84 | 53 | 137 | 10% | 35 | 7 | 43 | 51% |
| SVK | Slovakia | 1,300 | 94 | 32 | 125 | 10% | 1 | 1 | 2 | 2% |
| GHA | Ghana | 1,260 | 70 | 54 | 124 | 10% | 24 | 11 | 35 | 50% |
| PRT | Portugal | 350 | 73 | 49 | 122 | 35% | 1 | 1 | 3 | 3% |
| DOM | Dominican Republic | 1,260 | 91 | 21 | 111 | 9% | 4 | 2 | 6 | 7% |
| GBR | United Kingdom of Great Britain and Northern Ireland | 990 | 87 | 20 | 106 | 11% | 0 | 0 | 0 | 1% |
| PER | Peru | 19,370 | 71 | 29 | 100 | 1% | 2 | 0 | 2 | 3% |
| CHL | Chile | 5,710 | 85 | 14 | 99 | 2% | 5 | 3 | 8 | 9% |
| UGA | Uganda | 1,110 | 68 | 29 | 97 | 9% | 23 | 12 | 35 | 52% |
| CZE | Czechia | 1,100 | 60 | 26 | 86 | 8% | 1 | 1 | 1 | 2% |
| MOZ | Mozambique | 1,150 | 43 | 37 | 80 | 7% | 2 | 2 | 4 | 9% |
| KEN | Kenya | 930 | 59 | 19 | 78 | 8% | 13 | 6 | 19 | 32% |
| CHN | China | 2,800 | 64 | 12 | 76 | 3% | 2 | 2 | 3 | 5% |
| AUT | Austria | 1,210 | 58 | 18 | 75 | 6% | 0 | 1 | 1 | 1% |
| ITA | Italy | 2,740 | 45 | 30 | 75 | 3% | 2 | 1 | 3 | 7% |
| LTU | Lithuania | 660 | 51 | 20 | 71 | 11% | 0 | 1 | 1 | 2% |
| JPN | Japan | 5,450 | 50 | 19 | 70 | 1% | 3 | 2 | 5 | 10% |
| MMR | Myanmar | 3,530 | 50 | 18 | 68 | 2% | 1 | 1 | 2 | 4% |
| HUN | Hungary | 830 | 50 | 15 | 65 | 8% | 3 | 1 | 4 | 8% |
| COG | Congo | 3,760 | 40 | 24 | 64 | 2% | 1 | 2 | 3 | 8% |
| BLR | Belarus | 1,110 | 38 | 25 | 63 | 6% | 1 | 0 | 1 | 4% |
| CUB | Cuba | 510 | 10 | 54 | 63 | 12% | 0 | 0 | 0 | 5% |
| SWE | Sweden | 1,850 | 42 | 13 | 55 | 3% | 1 | 0 | 1 | 2% |
| GAB | Gabon | 4,800 | 39 | 13 | 52 | 1% | 1 | 1 | 2 | 4% |
| ETH | Ethiopia | 980 | 36 | 12 | 48 | 5% | 9 | 3 | 11 | 32% |
| BGR | Bulgaria | 2,120 | 36 | 12 | 48 | 2% | 2 | 3 | 5 | 13% |
| FIN | Finland | 1,260 | 33 | 14 | 47 | 4% | 2 | 1 | 3 | 10% |
| UKR | Ukraine | 810 | 33 | 14 | 47 | 6% | 2 | 1 | 2 | 7% |
| CAF | Central African Republic | 3,070 | 32 | 12 | 44 | 1% | 2 | 3 | 5 | 15% |
| GRC | Greece | 1,290 | 39 | 5 | 44 | 3% | 1 | 1 | 2 | 4% |
| IND | India | 2,650 | 31 | 14 | 44 | 2% | 2 | 3 | 5 | 16% |
| BEL | Belgium | 400 | 35 | 8 | 44 | 11% | 1 | 1 | 2 | 5% |
| SLE | Sierra Leone | 260 | 21 | 19 | 40 | 15% | 3 | 4 | 7 | 33% |
| MWI | Malawi | 160 | 27 | 9 | 36 | 23% | 0 | 1 | 1 | 5% |
| LVA | Latvia | 430 | 24 | 7 | 32 | 7% | 0 | 1 | 1 | 4% |
| HRV | Croatia | 990 | 21 | 9 | 31 | 3% | 0 | 0 | 0 | 1% |
| BLZ | Belize | 730 | 21 | 7 | 29 | 4% | 0 | 0 | 1 | 3% |
| LKA | Sri Lanka | 1,370 | 22 | 7 | 29 | 2% | 2 | 1 | 3 | 15% |
| SVN | Slovenia | 770 | 13 | 14 | 28 | 4% | 0 | 0 | 1 | 6% |
| ECU | Ecuador | 3,890 | 20 | 7 | 27 | 1% | 0 | 0 | 1 | 4% |
| GUF | French Guiana | 4,240 | 20 | 6 | 26 | 1% | 0 | 0 | 0 | 2% |
| ZWE | Zimbabwe | 70 | 17 | 9 | 26 | 37% | 0 | 0 | 0 | 2% |
| AGO | Angola | 410 | 21 | 4 | 25 | 6% | 0 | 0 | 1 | 4% |
| PAN | Panama | 1,330 | 15 | 8 | 23 | 2% | 0 | 1 | 1 | 6% |
| IRL | Ireland | 90 | 16 | 6 | 22 | 24% | 0 | 0 | 0 | 1% |
| CRI | Costa Rica | 1,020 | 10 | 10 | 20 | 2% | 0 | 0 | 1 | 7% |
| MYS | Malaysia | 1,970 | 0 | 20 | 20 | 1% | - | - | - | 0% |
| GIN | Guinea | 250 | 11 | 9 | 19 | 8% | 2 | 1 | 4 | 33% |
| PNG | Papua New Guinea | 980 | 12 | 6 | 18 | 2% | 0 | 1 | 1 | 12% |
| DNK | Denmark | 160 | 13 | 3 | 16 | 10% | 0 | 1 | 2 | 12% |
| PRI | Puerto Rico | 40 | 1 | 15 | 16 | 40% | 0 | 0 | 0 | 8% |
| MAR | Morocco | 220 | 11 | 3 | 15 | 7% | 0 | 0 | 0 | 4% |
| KOR | Korea, Republic of | 770 | 11 | 3 | 14 | 2% | 0 | 0 | 1 | 8% |
| EST | Estonia | 450 | 7 | 5 | 12 | 3% | 0 | 0 | 0 | 2% |
| TTO | Trinidad and Tobago | 140 | 11 | 1 | 12 | 9% | 0 | 0 | 0 | 3% |
| NLD | Netherlands | 160 | 9 | 2 | 11 | 7% | 0 | 0 | 0 | 2% |
| SRB | Serbia | 380 | 7 | 4 | 11 | 3% | 0 | 0 | 0 | 4% |
| JAM | Jamaica | 260 | 9 | 1 | 10 | 4% | 0 | 0 | 1 | 8% |
| DZA | Algeria | 40 | 3 | 6 | 9 | 23% | 0 | 0 | 0 | 4% |
| BGD | Bangladesh | 90 | 5 | 3 | 8 | 9% | 1 | 0 | 1 | 19% |
| DMA | Dominica | 20 | 0 | 6 | 7 | 35% | - | - | - | 0% |
| URY | Uruguay | 90 | 6 | 2 | 7 | 8% | 0 | 0 | 0 | 3% |
| TWN | Taiwan, Province of China | 640 | 6 | 0 | 6 | 1% | 0 | 0 | 1 | 9% |
| NPL | Nepal | 770 | 4 | 2 | 6 | 1% | 0 | 0 | 0 | 12% |
| GNQ | Equatorial Guinea | 440 | 4 | 2 | 5 | 1% | 0 | 0 | 0 | 12% |
| LUX | Luxembourg | 60 | 4 | 1 | 5 | 8% | 0 | 0 | 0 | 9% |
| NOR | Norway | 470 | 4 | 1 | 5 | 1% | 0 | 0 | 0 | 3% |
| SLV | El Salvador | 80 | 3 | 2 | 5 | 6% | 0 | 0 | 1 | 17% |
| SSD | South Sudan | 370 | 5 | 1 | 5 | 1% | 0 | 0 | 0 | 9% |
| ALB | Albania | 110 | 4 | 0 | 4 | 4% | 0 | 0 | 0 | 6% |
| HTI | Haiti | 30 | 1 | 3 | 4 | 13% | 0 | 0 | 0 | 8% |
| MTQ | Martinique | 50 | 3 | 0 | 3 | 6% | 0 | 0 | 0 | 6% |
| AZE | Azerbaijan | 250 | 1 | 0 | 2 | 1% | 0 | 0 | 0 | 29% |
| CHE | Switzerland | 110 | 1 | 0 | 2 | 2% | 0 | 0 | 0 | 3% |
| CYP | Cyprus | 50 | 1 | 1 | 2 | 4% | 0 | - | 0 | 1% |
| GNB | Guinea-Bissau | 10 | 1 | 1 | 2 | 20% | 0 | 0 | 0 | 27% |
| KAZ | Kazakhstan | 290 | 2 | 0 | 2 | 1% | 0 | 0 | 0 | 21% |
| LBR | Liberia | 270 | 1 | 1 | 2 | 1% | 0 | 0 | 0 | 2% |
| MKD | North Macedonia | 70 | 2 | 0 | 2 | 3% | 0 | 0 | 0 | 3% |
| REU | Réunion | 100 | 2 | 0 | 2 | 2% | 0 | 0 | 0 | 10% |
| TUN | Tunisia | 30 | 1 | 0 | 2 | 7% | - | - | - | 0% |
| BDI | Burundi | 50 | 1 | 0 | 1 | 2% | - | - | - | 0% |
| BHS | Bahamas | 10 | 0 | 0 | 1 | 10% | 0 | - | 0 | 0% |
| ISR | Israel | 10 | 1 | 0 | 1 | 10% | 0 | 0 | 0 | 14% |
| MNE | Montenegro | 30 | 1 | 0 | 1 | 3% | 0 | 0 | 0 | 3% |
| NCL | New Caledonia | 40 | 1 | 0 | 1 | 3% | 0 | 0 | 0 | 30% |
| RWA | Rwanda | 120 | 1 | 0 | 1 | 1% | 0 | 0 | 0 | 7% |
| TGO | Togo | 10 | 1 | 0 | 1 | 10% | 0 | 0 | 0 | 27% |
| **Total** |  | **631,570** |  |  | **25,460** | **4%** | **698** | **796** | **1,494** | **6%** |

Supplementary Analysis – Agricultural Suitability and Tree Cover Loss

One additional question we set out to answer was the degree to which agricultural suitability correlated with the likelihood of suitable land being converted from forest to other land use types. If a given protected forest was on land that was highly suitable for cropland, would that land be more likely to be converted for agricultural purposes?

We based this analysis on a cropland suitability raster dataset developed using multi-criteria analysis developed by Zabel et al. (2014) [28]. The dataset incorporated factors including climate, topography, and soil characteristics and represented the suitability for 16 major global crops.^[[2]](#footnote-2)^ Ranges for suitability varied between 0 and 100, which we reclassified into four categories according to guidance from [28]: 0 for not suitable, 1-33 for marginal, 34-74 for moderately suitable, and > 74 for highly suitable. We further refined this dataset to reclassify urban areas and water bodies (as classified in [20]) from suitable to non-suitable, with the assumption that these would not be used as agriculture (SI Figure 2). Our analysis incorporated several scenarios of IUCN protected areas, including one where we plotted the most stringently protected areas (classified as Ia, Ib, and II) as suitable and another variation that plotted all IUCN defined protected areas. We analyzed these datasets against the forest loss data [1] to derive where forest loss occurred and used ESA-CCI land cover data [20] to establish what land use change had occurred following the loss of forest land cover. This analysis was conducted for each individual PA to obtain the level of agricultural suitability for each area. All raster datasets were resampled to the same 30m resolution to enable harmonized analysis and we overlaid them using ArcGIS with a cylindrical equal area projection to retain true size of PAs throughout the global map.


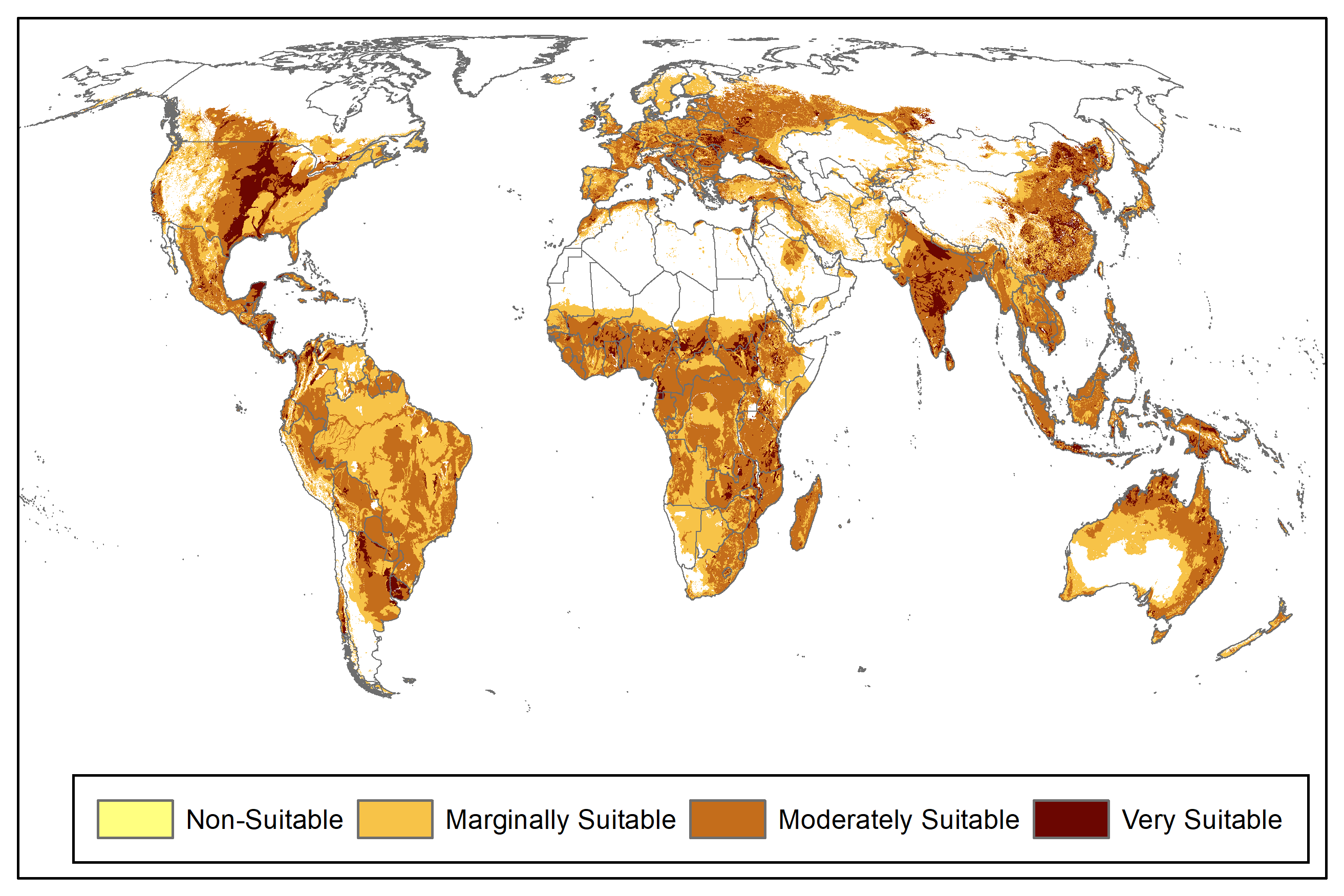


**Figure S2.** Agricultural suitability map.

We found that for protected forests; agricultural suitability, as a percentage of total land, was not a good indicator of agricultural conversion. Figure 3 demonstrates that there was a negligible difference in tree cover loss across the full spectrum of suitability of the protected area. If the proportion of land deemed suitable was a predictive indicator of tree cover loss, we would have expected to see a positive slope in the agricultural land cover class, with the percent of forest loss increasing as the proportion of land^[[3]](#footnote-3)^ suited to cultivate crops increased. Instead, we see no relationship.


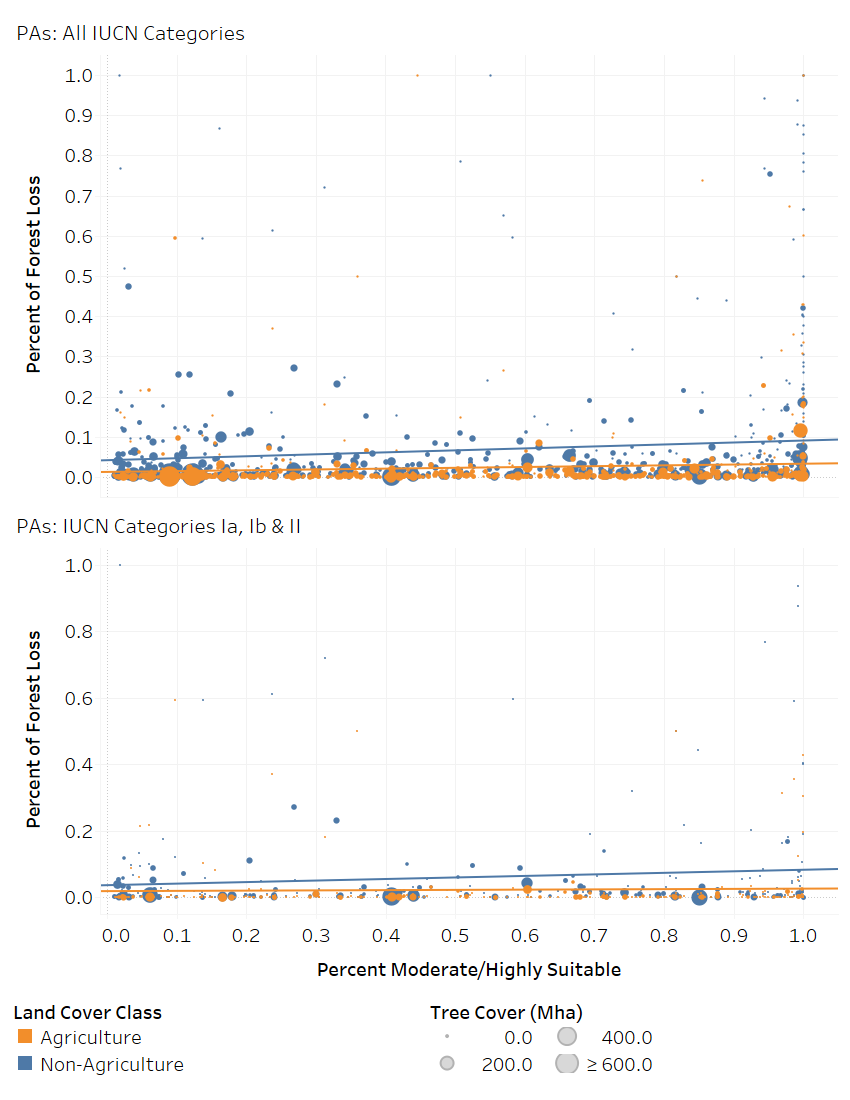


**Figure S3.** Relationship between agricultural suitability and land cover class following deforestation event within all IUCN categories (Top), and within more stringent IUCN categories (categories Ia, Ib, and II) (Bottom), shown as percent of tree cover within a PA lost between 2001-2014, where each dot is a PA.

Contrary to our expectations, we did not find a relationship between agriculturally driven forest loss and agricultural suitability^[[4]](#footnote-4)^, either in all PAs or in strict PAs (SI Figure 3). One potential explanation is that agriculture encroachment into PAs is not planned according to optimal suitability, but rather occurs in an opportunistic manner influenced by other factors such as access, cost of conversion, and likelihood of avoiding PA enforcement (e.g., situated farther from PA management offices). This explanation is supported by a study that found that deforestation within PAs in the tropics were more likely to be linked to small farms that were locally opportunistic, rather than large-scale agriculture that would be more strategic in citing operations [29].

While agricultural suitability data on a global scale might be useful to identify where there is suitability for cultivation, our analysis did not find a correlation between higher levels of agricultural suitability and higher incidents of forest loss and conversion to agriculture. There are further lines of inquiry to explore, however, that could inform the relationship between suitability and agricultural expansion in PAs. This includes, for example, refining suitability maps with locally specific information, or analyzing whether contagious deforestation occurs (e.g., parcels adjacent to suitable lands deforested for agriculture irrespective of their suitability category) [30, 31].

1. The summation across countries included in the table may not match to the Total listed due to rounding, and the exclusion of countries that did not experience tree cover loss within PAs from 2001 to 2018. [↑](#footnote-ref-1)
2. The study used criteria for 16 major crops including barley, cassava, corn, groundnut, millet, oil palm, potato, rapeseed, rice, rye, sorghum, soy, sugarcane, sunflower, summer wheat, and winter wheat. [↑](#footnote-ref-2)
3. Proportion of land suitable for crop cultivation from total protected area. [↑](#footnote-ref-3)
4. For this analysis we included cropland, mosaic cropland, and mosaic vegetation (which, by definition, contains less than 50% of mosaic cropland within each pixel) as agricultural lands. [↑](#footnote-ref-4)
